# Experimental Protocol to *Toxoplasma gondii* Detection in Fresh Goat Milk

**DOI:** 10.1155/2024/6895089

**Published:** 2024-06-26

**Authors:** Igor Falco Arruda, Patricia Riddell Millar, Mário Felipe Alvarez Balaro, Thamires Francisco Bonifácio, Raissa Cristina Ferreira Ramos, Maria Regina Reis Amendoeira

**Affiliations:** ^1^Toxoplasmosis and Other Protozoan Diseases Laboratory, Oswaldo Cruz Institute-Fiocruz, Rio de Janeiro, Brazil; ^2^Microbiology and Parasitology Department, Biomedical Institute-Fluminense Federal University, Niterói, Brazil; ^3^Experimental Research Unit in Goats and Sheep, Veterinary Medicine School-Fluminense Federal University, Cachoeiras de Macacu, Brazil

**Keywords:** fresh goat milk, goats, infectivity, survival, toxoplasmosis

## Abstract

*Toxoplasma gondii* is a zoonotic parasite with global distribution capable of infecting homeothermic animals. Transmission of protozoan to humans includes ingestion of water and raw food contaminated with sporulated oocysts, ingestion of raw or undercooked meat with tissue cysts, and tachyzoites' transplacental transmission. Fresh goat milk intake has already been linked to human toxoplasmosis outbreaks, but little is known about the infectious potential of this biological sample. Accordingly, the aim of the present study is to assess the survival and infectivity of *T. gondii* tachyzoites in fresh goat milk samples through an experimental protocol to detect this parasite via bioassay carried out with a murine model, DNA amplification, and serology. Swiss Webster mice were inoculated with fresh goat milk samples contaminated with different *T. gondii* RH strain tachyzoite concentrations per milliliter and stored for different refrigeration times. Animals showing clinical signs compatible to toxoplasmosis were euthanized. Milk samples contaminated with high parasitic loads and kept for a shorter refrigeration time were the most lethal ones. No significant differences were observed between mean death rates recorded for different goat milk contamination concentrations (*p* = 0.1888), and for the refrigeration time, contaminated milk samples were kept under (*p* = 0.9440). *T. gondii* DNA was amplified in all contaminated milk samples, but only one of the surviving mice was serologically positive. Results of the present study have shown *T. gondii* survival and infectivity in fresh goat milk samples, and it highlights its significant risk for public health. Therefore, molecular methods must be the tests of choice when milk samples are used to assess infection caused by protozoan in goats' dairy products.

## 1. Introduction

Toxoplasmosis is a zoonosis of great importance for public health. Protozoan *Toxoplasma gondii*, which is its etiologic agent, has felids as its definitive hosts; birds and mammals, including humans, are its intermediate hosts [1, 2]. Protozoan transmission occurs through three main pathways: sporulated oocysts ingestion through both water and raw food contaminated by cat feces in primary infection, ingestion of tissue cysts found in raw or undercooked meat of chronically infected animals, or, yet, through transplacental transmission [3, 4]. Some other less frequent mechanisms can account for *T. gondii* transmission to humans, with an emphasis on fresh goat milk consumption [5].

Goats are among the domestic species mostly sensitive to toxoplasmic infection [6], since toxoplasmosis is responsible for determining important reproductive changes, such as miscarriage, neonatal death, and mummified fetuses, in them [7]. Serological surveys carried out in Brazil, in the last 3 years, have detected different antibody frequencies in goat herds; it ranged from 21% to 46% [8–13]. In addition to damage caused to animal health, *T. gondii* infection in goatherds can have a significant economic impact for producers [14].

According to the literature, toxoplasmosis outbreaks in humans have already been linked to fresh goat milk consumption [15–17]. However, direct protozoan detection in this biological sample has been limited to parasitic DNA detection in the analyzed samples; however, their infectivity was not assessed [18–22]. The microscopic detection of free parasites in milk samples is not carried out because the identification of tachyzoites seems to be very difficult due to the large amount of fat droplets [6].

Therefore, given the scarcity of data on *T. gondii* viability goat milk and on the transmission potential of this biological sample, the aim of the current study was to assess the survival and infectivity of *T. gondii* tachyzoites in fresh goat milk samples, based on an experimental protocol to detect this parasite through bioassay carried out with a murine model, DNA amplification, and serology.

## 2. Material and Methods

### 2.1. Ethical Considerations

The present study was approved by the Ethics Committee on Animal Use (CEUA) of Oswaldo Cruz Institute, under license number L-041/2019.

### 2.2. Goat Milk Sample Obtainment and *T. gondii* Contamination

Approximately 1 L of fresh goat milk was collected from a Saanen goat bred and maintained at the Farm School of the Veterinary Medicine School of Fluminense Federal University to carry out the present experimental protocol. This site is located in Cachoeiras de Macacu City, Rio de Janeiro State, Brazil (22°31′13.5^″^S 42°42′28.3^″^W). The milk sample was obtained through manual milking, after goat's teat cleaning (predipping) and the discharge of the first two milk jets. Then, the sample was transported to the Toxoplasmosis and Other Protozoan Diseases Laboratory (LabTOXO) of Oswaldo Cruz Institute/Fiocruz, in sterile glass vials, in isothermal box, at a temperature close to 4°C. The milk sample was aliquoted in sterile 50-mL conical tubes and stored at −70°C, until processing, at LabTOXO. One of the aliquots was previously subjected to a conventional polymerase chain reaction (PCR) to amplify the repetitive element (REP) of 529 bp of *T. gondii* DNA [23] to confirm the absence of this protozoan's DNA in the collected sample.

Tachyzoites of *T. gondii* RH strain kept in female Swiss Webster mice and obtained through peritoneal lavage were used to contaminate 30-mL aliquots of goat milk. These aliquots were contaminated at the following concentrations: 5 × 10^5^, 5 × 10^4^, 5 × 10^3^, 5 × 10^2^, and 5 × 10^1^ parasites/milliliter. Initial concentration (5 × 10^5^) was chosen because it is standardized in the laboratory, in the protocol to maintain the strain in the murine model. The contaminated samples, as well as an aliquot of noncontaminated milk, were stored and kept for 24, 48, 72, and 96 h under refrigeration (2°C–8°C). Milk samples were concentrated and resuspended in 2 mL of 1% PBS, supplemented with 2% antibiotic (1000 UI penicillin + 100 *μ*g streptomycin/milliliter) for 4-day postcontamination, on a daily basis. In total, 1.5 mL of this volume was destined to assess tachyzoites' survival time and infectivity in contaminated milk; the remaining 0.5 mL was used to confirm the presence of parasite DNA in the sample.

### 2.3. Assessing *T. gondii* Tachyzoites' Survival and Infectivity in Goat Milk Samples

Concentrated 1.5-mL aliquots were intraperitoneally inoculated in groups of three female Swiss Webster mice, in the age group 25–30 days, divided based on each contamination concentration and on time interval under refrigeration, to assess *T. gondii* tachyzoites' survival time and infectivity in contaminated milk samples. Each animal was inoculated with 0.5 mL of the concentrated milk sample. Inoculated mice were followed up on a daily basis for 60 days, at most. Animals presenting clinical signs compatible to acute toxoplasmosis, such as prominent piloerection, dyspnea, chest breathing, and ascites, were euthanized. The sick animals were restrained manually using physical restraint. Initially, the animal was pressed lightly onto the surface of the cage grid and held by the skin of the dorsocervical region, between the index and thumb fingers, clamping its tail between the other fingers and the palm of the hand, to completely limit its movements. In this way, the right hind limb was extended to facilitate visualization and access to the biceps femoris muscle. After the immobilization and asepsis of this anatomical region, 300 *μ*L of anesthetic overdose associating ketamine hydrochloride (300 mg/kg) and xylazine hydrochloride (30 mg/kg) was administered intramuscularly, using a 1 mL syringe and a 13 × 0.3 mm needle, to implement a humane endpoint [24]. Free tachyzoites were surveyed on slides with 10 *μ*L of peritoneal lavage to confirm parasites' survival and infectivity.

### 2.4. Detecting Parasite DNA in Goat Milk Samples Contaminated With *T. gondii* Tachyzoites

Aliquots of 0.5 mL of milk were subjected to molecular analysis to confirm the presence of tachyzoites in samples contaminated with different concentrations and kept under different refrigeration time intervals. Initially, the concentrated samples were washed three times in 2 mL of UltraPure DNase/RNase-Free Distilled Water, Invitrogen®, to remove extraction inhibitors and to purify the DNA [25]. Then, 200 *μ*L of concentrated milk was used for DNA extraction in commercial QIAamp DNA Blood Mini Kit, Qiagen® (250), according to the manufacturer's specifications. The extracted DNA was used to amplify the 529 bp REP of the *T. gondii* genome through PCR, by using primers TOX4 (CGCTGCAGGGAGGAAGACGAAAGTTG) and TOX5 (CGCTGCAGACACAGTGCATCTGGATT) [23].

Each performed reaction that used a final volume of 25 *μ*L of PCR mix was added with 14 *μ*L ultrapure water, 4 *μ*L dNTP (1.25 mM), 2.5 *μ*L buffer (10X), 0.75 *μ*L MgCl2 (50 mM), 0.5 *μ*L TOX4 (10 pmol/*μ*L), 0.5 *μ*L TOX5 (10 pmol/*μ*L), 0.25 *μ*L Platinum Taq DNA polymerase (Invitrogen®), and 2.5 *μ*L extracted DNA. PCR mixes were subjected to initial denaturation at 94°C, for 7 min, and to 35 cycles at 94°C, for 1 min (denaturation), at 55°C for 1 min (annealing), at 72°C for 1 min (extension), and to final extension at 72°C for 10 min. The produced amplicons were detected through electrophoresis, in 1.5% agarose gel, stained with *GelRed Nucleic Acid Gel Stain* (Biotium®), and visualized under ultraviolet light in a transilluminator.

### 2.5. Detecting IgG Anti-*T. gondii* Antibodies in Surviving Mice

Surviving mice underwent cardiac puncture, followed by euthanasia, to collect approximately 1 mL of blood for IgG anti-*T.gondii* antibodies, 60 days after inoculation. Blood samples were centrifuged at 1000 × g for 10 min to collect the serum. Sera were subjected to an indirect fluorescent antibody test (IFAT) [26]. *T. gondii* tachyzoite RH strain inactivated in 2% formalin was used as antigen. Commercial anti-Mouse IgG (whole molecule)-FITC conjugate produced in goats (Sigma-Aldrich®) diluted in Evans Blue solution was used to detect anti-*T. gondii* IgG. Samples were considered positive when tachyzoites' total surface fluorescence was observed at a dilution ratio equal to, or higher than, 1 : 16.

### 2.6. Statistical Analysis

Daily inoculated mice follow-ups were recorded in Microsoft Excel® spreadsheets, from inoculation day onwards. The quantitative variable included in these spreadsheets was the number of deaths observed or euthanasias implemented per day postinoculation in the animals from the different groups inoculated with contaminated milk samples. The qualitative result regarding the presence or absence of tachyzoites in the peritoneal lavage performed was also recorded on these spreadsheets. Simple ANOVA was performed in GraphPad Prism®, version 5 to assess the difference between mean death values in relation to contamination concentration with parasites and refrigeration time. Values ≤ 0.05 were considered significant.

## 3. Results

**Figure 1:**
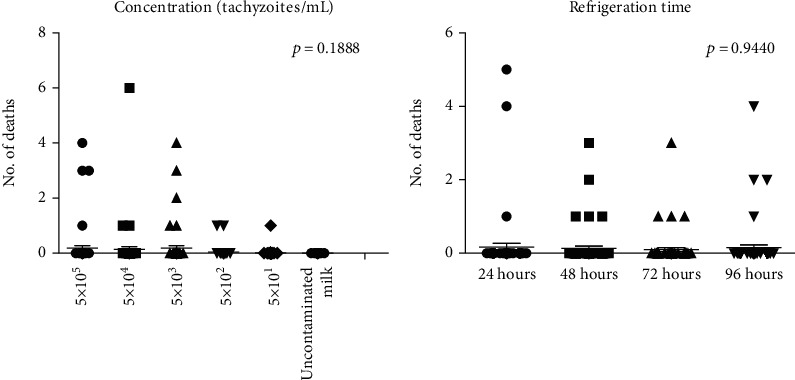
Distribution of number of deaths of Swiss Webster mice based on inoculated milk contamination concentration and refrigeration time.

In total, 45.8% (33/72) of the total number of inoculated animals died or were euthanized, because they manifested clinical signs of acute toxoplasmosis, including symptoms such as noticeable piloerection, dyspnea, antalgic position, and ascites. The highest rate of deaths/euthanasia, 90.9% (30/33), was recorded between the 6^th^ and 9^th^ day after inoculation (Table 1). Milk samples contaminated with 5 × 10^5^ and 5 × 10^3^ tachyzoites/milliliter were the most lethal ones; each of them accounted for the death of 11 animals. Contaminated milk samples kept under refrigeration for 24 h were responsible for the death of 10 animals. No significant differences were observed between mean death rates among different goat milk contamination concentrations (*p* = 0.1888), and for refrigeration times, contaminated milk samples were kept under (*p* = 0.9440).  Figure 1 shows the distribution of the number of deaths caused by different contamination concentrations, and for refrigeration time, goat milk samples were kept under. Free tachyzoites were observed in the peritoneal lavage samples taken from animals found dead or euthanized.

The molecular analysis based on the amplification of the 529 bp fragment confirmed the presence of *T. gondii* DNA in all goat milk samples, regardless of the inoculated concentration and refrigeration time.

Only one of the 39 surviving mice was considered seropositive for IgG anti-*T. gondii* by IFAT. This surviving animal was inoculated with goat milk sample contaminated with 5 × 10^5^ parasites/milliliter and kept under refrigeration for 72 h; it showed fluorescence of tachyzoite surface at a dilution ratio of 1 : 16.

All mice inoculated with uncontaminated milk survived for 60 days after inoculation; they showed no clinical changes at follow-up time and remained seronegative for anti-*T. gondii* antibodies. In addition, all uncontaminated milk samples were negative in PCR, regardless of refrigeration time.

## 4. Discussion

Results in the present study have shown that *T. gondii* tachyzoites can remain infective and viable in fresh goat milk samples under refrigeration (2°C–8°C) for 96 h (4 days), after sample collection. Walsh et al. [27] observed higher values when they assessed *T. gondii* tachyzoite infectivity, for 168 h (7 days), in contaminated goat milk samples kept at 4°C, in a model in vitro. Walsh et al. [27] used *T. gondii* RH strain to assess protozoan infectivity in experimentally contaminated goat milk samples. *T. gondii* RH strains belong to clonal lineage type 1; they presented high virulence, low lethal dose in a murine model, and low tachyzoite–bradyzoite interconversion [28]. Assumingly, the high intrinsic infectivity of this strain has contributed to this protozoan survival, as well as to its infectivity in milk samples kept under refrigeration for longer periods of time. This process may not be the case of clonal type 2 and 3 strains, as well as of nonclonal strains. Further studies focused on assessing the survival and infectivity of *T. gondii* tachyzoites of other genotypes in goat milk samples are necessary.

It is worth noticing that fresh goat milk's short perishability can be an important challenge for attempts to isolate *T. gondii* in samples from naturally infected animals whose samples' parasite load is unknown. Therefore, samples should be inoculated in murine models as soon as possible (few hours after collection) and kept under refrigeration (from 2°C to 8°C) to increase the possibility of *T. gondii* isolation from goat milk, as well as to assess its infectivity and virulence. This protocol would be the most favorable one to assess optimal tachyzoite viability [29]. This finding can be corroborated by the infectivity observed in the herein assessed goat milk samples contaminated with low tachyzoite concentrations (5 × 10^1^ parasites/milliliter) and under refrigeration for 24 h.

*T. gondii* tachyzoites' infectivity in fresh goat milk samples may also be related to contamination concentration and to the protozoan strain used in the protocol. Milk samples contaminated with up to 5 × 10^3^ tachyzoites/milliliter of *T. gondii* RH strain were the most lethal ones for the inoculated mice (with euthanasia implemented in 14 instances) in the current study. Dubey et al. [30] observed a correlation between goat milk samples' contamination concentration and parasite infectivity. Clinical signs of toxoplasmosis were identified in mice inoculated with goat milk samples contaminated with up to 1.6 × 10^−3^ tachyzoites of *T. gondii* GT-1 strain, clonal type 1 [30]. In addition to genotype, the parasite load found in the sample can contribute to infectivity, as well as to the manifestation of the acute disease in inoculated mice. However, little is known about the approximate amount of parasites shed in milk samples from naturally infected goats. Molecular analyses have evidenced that goats naturally or experimentally exposed to this protozoan can eliminate parasite DNA in milk; however, these analyses did not assess the infective potential of this biological sample [18, 30]. In short, studies strongly supporting the isolation of viable forms of *T. gondii* from goat milk remain scarce in the literature [6].

Bioassay in a murine model (outbred Swiss Webster mice) was the method of choice to assess *T. gondii* infectivity in goat milk. The use of outbred mice in infectivity assays carried out with *T. gondii* strains allows determining the degree of virulence of parasite isolates [31, 32]. Other methods, such as inoculation of milk samples in cell culture, also make it possible assessing the infective potential of goat milk in transmitting this protozoan [27]. However, *T. gondii* isolation in cell culture can present some disadvantages, such as the possibility of cells' contamination and greater sensitivity of cells in vitro to the components of tested biological samples, in comparison to models tested in vivo [29]. In addition, the isolation of *T. gondii* in cell culture does not allow us to determine the degree of virulence of the isolates, measured by mortality and morbidity values, when positive [33, 34]. The determination of virulence associated with the identification of the genotype of naturally circulating strains of *T. gondii* is necessary to establish the phenotypic and genotypic profiles of the isolates, as well as their potential to determine symptomatic cases of toxoplasmosis. However, alternative methods to assess *T. gondii* strains' infectivity and virulence are extremely necessary, since they aim at refining, reducing, or even replacing the use of murine models, from the 3R perspective.

In this study, the route of infection used in the experimental protocol was intraperitoneal. Dubey et al. [30] evaluated the survival of *T. gondii* tachyzoites in contaminated goat's milk by means of subcutaneous inoculations in mice. On the other hand, the infectivity of *T. gondii* present in artificially contaminated milk samples can also be achieved through oral administration in a murine model [35]. The choice of the intraperitoneal route in this protocol, rather than the oral route, was due to the greater sensitivity of tachyzoites to gastric juice [36]. Furthermore, this study is aimed at assessing the survival and infectivity of tachyzoites in fresh goat's milk and not the potential for parasite transmission from this biological sample, which would require a simulation of the natural transmission route. Considering that tachyzoites are the parasitic forms present in the extracellular environment and in body fluids, the choice of the intraperitoneal route of infection aimed to preserve the survival of as many parasites as possible in goat milk samples. However, Dubey [37] observed a certain degree of survival and infectivity of tachyzoites of *T. gondii* RH strain exposed to acid pepsin solutions in a murine model orally infected. In this sense, the protocol presented here should be tested in the future to assess the infectivity of tachyzoites present in fresh goat's milk by means of oral inoculations.

Most mice in the present study were seronegative for IgG anti-*T. gondii*. Dubey et al. [30] did not observe seroconversion in mice inoculated with goat milk contaminated with low *T. gondii* GT1 strain tachyzoite (10^−4^ to 10^−6^) concentrations. However, IgG anti-*T. gondii* antibodies have been detected 14 days after inoculation, onwards, in serum samples from outbred OF1 mice who were orally infected with tissue cysts of *T. gondii* 76 K strain [38]. Several factors can influence virulence assessment and, consequently, the kinetics of antibody production and secretion in infection caused by *T. gondii* in murine models, such as infecting dose, parasite strain, and evolutionary form used for infection [31]. The use of *T. gondii* RH strain in the current experiment can explain the absence, or low production, of antibodies against this parasite among inoculated mice. The RH strain became unable to naturally produce tissue cysts in the murine model after successive intraperitoneal passages in mice, in a laboratory environment [39]. Tissue cyst formation appears to be a key event to produce serum IgG antibodies during *T. gondii* infection [40]. Parasite persistence in tissues can act as a continuous stimulus for humoral immune response. Therefore, the acute nature of infection caused by *T. gondii* RH strain, which presents a lethal profile in mice within 3–7 days after inoculation [31], may be too accelerated to trigger antibody production.

However, a single serum sample from a mouse inoculated with contaminated milk was positive in IFAT. Despite the high inoculated parasite load (5 × 10^5^), this animal survived the experimental protocol, as well as produced antibodies against this parasite, although at low titers (1 : 16). Intrinsic factors linked to this animal were assumingly linked to its survival; this finding points out the need of further studies to analyze the association among parasite load, seroconversion, and immunogenic susceptibility of the host. However, the inoculated parasite load seemed to be directly related to the seroconversion of the analyzed mice. Seronegativity and survival of mice herein inoculated with milk contaminated with lower amounts of tachyzoites could be explained by the control of inoculated parasite forms based on animals' immune response. Thus, lack of antigenic stimulus may have compromised antibodies' production. Therefore, the use of serological methods to indirectly detect *T. gondii* in serum samples from mice inoculated with fresh goat milk should be assessed with caution because parasite strains presenting virulent behavior or low cystogenic capacity and unknown concentrations of eventual parasites in the milk may not induce a humoral response in murine models.

REP 529 bp amplification through conventional PCR showed the best *T. gondii* detection results in the present study among all methods applied to parasite detection in contaminated goat milk samples. Molecular detection of *T. gondii* in milk samples of goats naturally exposed to the parasite has been widely used in Brazil and in other countries, such as Italy, Tunisia, and Poland based on the adoption of different gene targets of this parasite, as well as on conventional PCR on its variations [41–44]. Dubey et al. [30] observed the best performance of the nested PCR (nPCR) model in comparison to the conventional PCR model in the molecular detection of *T. gondii* in intentionally contaminated goat milk samples and to experimentally infected animals. In the current study, conventional PCR was able to amplify REP 529 bp even in samples contaminated with the lowest concentration of parasites in the milk. However, it is worth noticing that little is known about the quantification of parasite forms or of DNA excreted in milk by goats exposed to *T. gondii*. Therefore, despite the successful detection of DNA from this protozoan parasite in all the analyzed contaminated milk samples, future studies employing more sensitive molecular methods, such as the nPCR model, can be more effective in detecting goats naturally exposed to this protozoan parasite.

Finally, results in the present study evidenced the survival and infectivity of *T. gondii* RH strain tachyzoites in fresh goat milk samples at 96-h refrigeration, mainly at high concentrations of this parasite. This phenomenon, although observed in an experimental model, highlights its significant risk for public health, mainly for populations used to consume fresh goat milk. Goat milk should be inoculated in a murine model, 24 h after sample collection, and kept under refrigeration until processing time in a laboratory environment to increase the possibility of *T. gondii* isolation from this biological sample. However, molecular detection of *T. gondii* in goat milk seems to be the most effective direct method to detect the parasite in these samples, because, besides not requiring immediate processing, it can more accurately identify goats naturally exposed to this parasite. The indirect detection of *T. gondii* through the serology of mice inoculated with milk does not seem to be an effective method to identify the presence of this parasite, or its components, in goat milk, mainly when one is dealing with strains of unknown biological behavior that circulate naturally among goat herds.

## Figures and Tables

**Table 1:** Distribution of Swiss Webster mice inoculated with goat milk samples contaminated with different *T. gondii* tachyzoite concentrations, kept under different refrigeration times, by days of death.

**Refrigeration time**	**Concentration of milk contamination (parasites/milliliter)**	**Days (# of deaths/# of total group)**																			
		**1**	**2**	**3**	**4**	**5**	**6**	**7**	**8**	**9**	**10**	**11**	**12**	**13**	**14**	**15**	**16**	**17**	**18**	**19**	**≥ 20**
24 h	5 × 10^5^	0/3	0/3	0/3	0/3	0/3	3/3^a^														
	5 × 10^4^	0/3	0/3	0/3	0/3	0/3	1/3^a^	2/2^a^													
	5 × 10^3^	0/3	0/3	0/3	0/3	0/3	0/3	3/3^a^													
	5 × 10^2^	0/3	0/3	0/3	0/3	0/3	0/3	0/3	0/3	0/3	0/3	0/3	0/3	0/3	0/3	0/3	0/3	0/3	0/3	0/3	0/3
	5 × 10^1^	0/3	0/3	0/3	0/3	0/3	0/3	0/3	0/3	1/3^a^	0/2	0/2	0/2	0/2	0/2	0/2	0/2	0/2	0/2	0/2	0/2
	Uncontaminated milk	0/3	0/3	0/3	0/3	0/3	0/3	0/3	0/3	0/3	0/3	0/3	0/3	0/3	0/3	0/3	0/3	0/3	0/3	0/3	0/3

48 h	5 × 10^5^	0/3	0/3	0/3	0/3	0/3	1/3	1/2	0/1	1/1											
	5 × 10^4^	0/3	0/3	0/3	0/3	0/3	0/3	1/3	0/2	0/2	0/2	0/2	0/2	0/2	0/2	0/2	0/2	0/2	0/2	0/2	0/2
	5 × 10^3^	0/3	0/3	0/3	0/3	0/3	0/3	1/3^a^	2/2^a^												
	5 × 10^2^	0/3	0/3	0/3	0/3	0/3	0/3	0/3	0/3	0/3	0/3	0/3	0/3	0/3	0/3	1/3	0/2	0/2	0/2	0/2	0/2
	5 × 10^1^	0/3	0/3	0/3	0/3	0/3	0/3	0/3	0/3	0/3	0/3	0/3	0/3	0/3	0/3	0/3	0/3	0/3	0/3	0/3	0/3
	Uncontaminated milk	0/3	0/3	0/3	0/3	0/3	0/3	0/3	0/3	0/3	0/3	0/3	0/3	0/3	0/3	0/3	0/3	0/3	0/3	0/3	0/3

72 h	5 × 10^5^	0/3	0/3	0/3	0/3	0/3	0/3	1/3^a^	1/2	0/1	0/1	0/1	0/1	0/1	0/1	0/1	0/1	0/1	0/1	0/1	0/1
	5 × 10^4^	0/3	0/3	0/3	0/3	0/3	0/3	0/3	0/3	1/3	0/2	0/2	0/2	0/2	0/2	0/2	0/2	0/2	0/2	0/2	0/2
	5 × 10^3^	0/3	0/3	0/3	0/3	0/3	0/3	0/3	1/3	0/2	0/2	1/2	0/1	0/1	0/1	0/1	0/1	0/1	0/1	0/1	0/1
	5 × 10^2^	0/3	0/3	0/3	0/3	0/3	0/3	0/3	1/3	0/2	0/2	0/2	0/2	0/2	0/2	0/2	0/2	0/2	0/2	0/2	0/2
	5 × 10^1^	0/3	0/3	0/3	0/3	0/3	0/3	0/3	0/3	0/3	0/3	0/3	0/3	0/3	0/3	0/3	0/3	0/3	0/3	0/3	0/3
	Uncontaminated milk	0/3	0/3	0/3	0/3	0/3	0/3	0/3	0/3	0/3	0/3	0/3	0/3	0/3	0/3	0/3	0/3	0/3	0/3	0/3	0/3

96 h	5 × 10^5^	0/3	0/3	0/3	0/3	0/3	0/3	1/3^a^	2/2												
	5 × 10^4^	0/3	0/3	0/3	0/3	0/3	0/3	3/3^a^													
	5 × 10^3^	0/3	0/3	0/3	0/3	0/3	0/3	0/3	0/3	2/3	0/1	0/1	0/1	1/1							
	5 × 10^2^	0/3	0/3	0/3	0/3	0/3	0/3	0/3	0/3	0/3	0/3	0/3	0/3	0/3	0/3	0/3	0/3	0/3	0/3	0/3	0/3
	5 × 10^1^	0/3	0/3	0/3	0/3	0/3	0/3	0/3	0/3	0/3	0/3	0/3	0/3	0/3	0/3	0/3	0/3	0/3	0/3	0/3	0/3
	Uncontaminated milk	0/3	0/3	0/3	0/3	0/3	0/3	0/3	0/3	0/3	0/3	0/3	0/3	0/3	0/3	0/3	0/3	0/3	0/3	0/3	0/3

^a^Euthanasia implementation.

## Data Availability

The data that support the findings of this study are available from the corresponding author upon request.
